# Molecular Spring Enabled High-Performance Anode for Lithium Ion Batteries

**DOI:** 10.3390/polym9120657

**Published:** 2017-11-29

**Authors:** Tianyue Zheng, Zhe Jia, Na Lin, Thorsten Langer, Simon Lux, Isaac Lund, Ann-Christin Gentschev, Juan Qiao, Gao Liu

**Affiliations:** 1 Energy Storage and Distributed Resources Division, Lawrence Berkeley National Laboratory, 1 Cyclotron Rd., Berkeley, CA 94720, USA; tyzheng@lbl.gov (T.Z.); zhejia15@gmail.com (Z.J.); 2 Key Lab of Organic Optoelectronics and Molecular Engineering of Ministry of Education, Department of Chemistry, Tsinghua University, Beijing 100084, China; nalin_2016@163.com (N.L.); qjuan@mail.tsinghua.edu.cn (J.Q.); 3 BMW Group, Petuelring 130, 80788 Munich, Germany; Thorsten.Langer@bmw.de; 4 BMW Group Technology Office USA, 2606 Bayshore Parkway, Mountain View, CA 94043, USA; Simon.Lux@bmw.de (S.L.); Isaac.Lund@bmw.de (I.L.); Ann-Christin.Gentschev@bmw.de (A.-C.G.)

**Keywords:** energy storage, lithium-ion battery, conductive polymer binder, silicon/graphene, molecular spring, high loading

## Abstract

Flexible butyl interconnection segments are synthetically incorporated into an electronically conductive poly(pyrene methacrylate) homopolymer and its copolymer. The insertion of butyl segment makes the pyrene polymer more flexible, and can better accommodate deformation. This new class of flexible and conductive polymers can be used as a polymer binder and adhesive to facilitate the electrochemical performance of a silicon/graphene composite anode material for lithium ion battery application. They act like a “spring” to maintain the electrode mechanical and electrical integrity. High mass loading and high areal capacity, which are critical design requirements of high energy batteries, have been achieved in the electrodes composed of the novel binders and silicon/graphene composite material. A remarkable area capacity of over 5 mAh/cm^2^ and volumetric capacity of over 1700 Ah/L have been reached at a high current rate of 333 mA/g.

## 1. Introduction

Lithium-ion batteries (LIBs) have emerged as a critical electrical energy storage solution in consumer electronics, transportation and stationary storage [1,2,3]. The lithium-ion battery electrodes consist of nano to micron-sized ceramic particles for lithium ion storage, which are laminated together with a few percent of polymer binders. These electrode laminates need to be both electronically and ionically conductive throughout the battery lifetime. The degradation of the adhesive properties of the polymer in the electrode causes the decay of the both the mechanical and electrically integrity of the electrode, leading to battery capacity fade. Therefore, the polymer binders play a key role in the electrode formulation. Moreover, the current quest for higher energy density and longer battery operational lifetime has driven the development of new multifunctional polymer binder materials for emerging new energy storage chemistry.

State-of-the-art LIBs utilize graphite as an anode material with a theoretical specific capacity of 372 mAh/g and a stable electrochemical performance [4,5,6]. However, the search for materials with higher capacity leads to silicon, a naturally abundant material, which possesses the highest specific capacity (theoretically 4200 mAh/g) among any lithium-ion anode materials. However, Si has a large volume change (~300%) between lithiated and delithiated states, destabilizing both the Si materials itself and the composite electrode made with Si materials [7,8,9]. Numerous research efforts have been devoted to revealing the mechanism of electrode collapse and capacity fading of Si materials so as to make their practical usage for LIBs [10,11,12]. As a result, there are recent surged activities to develop new multifunctional binders for Si-containing electrodes [13,14,15]. To enhance the cycling stability of silicon electrodes and better accommodate volume swing of the Si materials, electrically conductive polymers have been developed and used as binders to integrate the active materials in the electrode matrix [16,17]. However, most of the conductive polymers are not developed for adhesive applications. Although they have good conductivity due to the conjugated backbones, they are very rigid and not suitable as an adhesive, nor is it easy to incorporate new functional groups to modify their properties. A new class of conductive polymer binders based on a methacrylate backbone and a pyrene side chain moiety has been developed and demonstrated superb performance as polymer binder materials for lithium-ion anode applications [18]. This class of conductive polymer can be synthesized via radical polymerization process of the methacrylate moiety. The monomers are simple to synthesize, and the polymerization process can tolerate wide range of functional groups. Further synthesis of different analogies of these polymers provides a library of binders to catering different needs for advanced lithium-ion battery applications [19,20,21].

**Figure 1:**
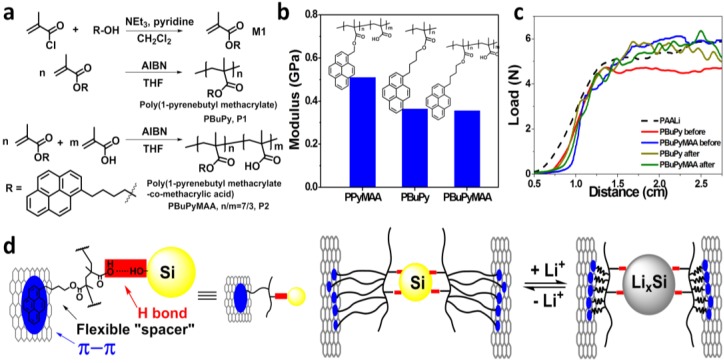
(**a**) The synthesis to Poly(1-pyrenebutyl methacrylate) (PBuPy) and Poly(1-pyrenebutyl methacrylate-co-methacrylic acid) (PBuPyMAA); (**b**) Modulus of PPyMAA [19], PBuPy and PBuPyMAA polymer binders, with their structures inserted. Adapted with permission from [19], American Chemical Society, 2015; (**c**) Peel test on PBuPy and PBuPyMAA laminates before and after cycling; (**d**) Mechanism of the polymer acts as a molecular spring to maintain the electrode integrity during the volume change of silicon.

In this work, we developed two new conductive polymer binders—Poly(1-pyrenebutyl methacrylate) (PBuPy) and Poly(1-pyrenebutyl methacrylate-*co*-methacrylic acid) PBuPyMAA (structures are shown in Figure 1), in which butyl segments are synthetically incorporated between the methacrylate chain and the pyrene moiety. The two polymer binders are characterized to be flexible to maintain the electrode integrity during Si volume change, enabling high capacity and good cycling stability for silicon containing electrodes. An area capacity of over 5 mAh/cm^2^ and volumetric capacity of over 1700 Ah/L, and stable cycling at this capacity have been achieved with these new binders and a Si/graphene composite material at high mass loading.

## 2. Materials and Methods 

Otherwise stated, all the chemicals are purchased from Sigma-Aldrich (St. Louis, MO, USA) and used without further treatment. The silicon/graphene composite material and the graphene are provided by XG Sciences (Lansing, MI, USA). Proton nuclear magnetic resonance (^1^H-NMR) spectra were recorded by using Avance II 500 MHz NMR Spectrometer (Bruker Corporation, Billerica, MA, USA) at The Molecular Foundry, in deuterated solvent with tetramethylsilane (TMS) as internal reference; chemical shifts (δ) are reported in parts per million. Matrix-assisted laser desorption ionization time-of-flight (MALDI-TOF) mass spectra were measured on a 4800 MALDI TOF-TOF analyzer (Applied Biosystems, Foster City, CA, USA) at The Molecular Foundry (dithranol as matrix). Fourier-transform infrared spectroscopy (FT-IR) spectra were recorded on a Bomem MB102 spectrometer (ABB, Quebec, QC, Canada) from 400 to 4000 cm^−1^. Molecular weights and distributions of polymers were measured by Gel permeation chromatography (GPC) with tetrahydronfuran (THF) eluent and polystyrene standard, using a Waters Associates liquid chromatography equipped with a Waters 510 HPLC pump and a Waters 2998 PDI detector (all from Waters Corporation, Milford, MA, USA). Thermal gravimetric analysis (TGA) was performed up to 600 °C at a SDT Q600 TGA/DSC system (TA Instruments, New Castle, DE, USA). Nano-indentation test was performed on a Hysitron TI900 TriboIndenter (Bruker Corporation, Billerica, MA, USA), calibrated with aluminum. The 1-Pyrenebutyl methacrylate (M1), PBuPy (P1) and PBuPyMAA (P2) are synthesized according to reported procedures with relative starting materials.

*Synthesis of 1-Pyrenebutyl methacrylate* (*M1*). 1-Pyrenebutanol (6.0 g, 21.9 mmol) was dissolved in anhydrous CH_2_Cl_2_ (120 mL). At 0 °C, triethylamine (12 mL) and pyridine (5 mL) were added, and the mixture was kept stirring for 30 min. Then methacryloyl chloride (8.8 g, 84.2 mmol) was added dropwise and the mixture was stirred for another 1 h at room temperature. After that, the reaction was quenched by water (75 mL) and extracted with CH_2_Cl_2_. The extract was washed with aqueous HCl (1 M, 150 mL), aqueous NaHCO_3_ (5%, 150 mL), and brine (150 mL). The solvent was removed and the crude product was recrystallized with methanol to obtain the final product as white powder (4.8 g, 13.9 mmol, 64%). ^1^H-NMR (500 MHz, CDCl_3_): δ 8.27 (d, *J* = 9.5 Hz, 1H), 8.17 (m, 2H), 8.11 (m, 2H), 8.03 (d, *J* = 2 Hz, 2H), 7.99 (m, 1H) 7.87 (d, *J* = 8 Hz, 1H), 6.09 (s, 1H), 5.54 (s, 1H), 4.23 (t, *J* = 6.5 Hz, 2H), 3.40 (t, *J* = 7.8 Hz, 2H), 1.97 (m, 2H), 2.00 (s, 3H), 1.85 (m, 2H) ppm. MALDI-TOF MS: cald. 342.44, Found 342.07.

*Synthesis of Poly(1-pyrenebutyl methacrylate)* (*PBuPy*, *P1*)*.* 1-Pyrenebutyl methacrylate (1.0 g, 2.9 mmol) was dissolved in freshly distilled THF (10 mL). To the solution, 2,2′-azobis(2-methyl propionitrile) (AIBN) (9.7 mg, 0.06 mmol) was added. The mixture was degassed by three freeze-evacuate-thaw cycles and heated to 60 °C for 24 h. The product was purified by precipitation with diethyl ether. (Product: 0.9 g, 90%) ^1^H-NMR (500 MHz, CDCl_3_): 0.98–1.50 (br, 7H), 1.98 (br, 2H), 2.81 (br, 2H), 3.83 (br, 2H), 7.66–7.83 (br, 9H) ppm. GPC: M_n_ = 7.1 kDa, PDI = 2.9.

*Synthesis of Poly(1-pyrenebutyl methacrylate-co-methacrylic acid)* (*PBuPyMAA*, *P2*)*.* 1-Pyrenebutyl methacrylate (1.0 g, 2.9 mmol) and methacrylic acid (0.11 g, 1.3 mmol) (were dissolved in freshly distilled THF (20 mL). To the solution AIBN (14 mg, 0.09 mmol) was added. The mixture was degassed by three freeze-evacuate-thaw cycles and heated to 60 °C for 24 h. The product was purified by precipitation with diethyl ether. (Product: 1.0 g, 90%) ^1^H-NMR (500 MHz, DMSO): δ 1.08–1.50 (br, 7H), 1.99 (br, 2H), 2.79 (br, 2H), 3.80 (br, 2H), 7.77 (br, 9H), 12.80 (br, 0.4H) ppm. GPC: M_n_ = 10.6 kDa, PDI = 2.8.

The adhesion strength of the laminate was measured by peel test at a Chatillon^®^ TCD225 series force measurement system (AMETEK, Largo, FL, USA). The Cu side of the electrode (1.2 cm × 1.2 cm) was fixed vertically to the bottom sample holder. The adhesive side of 3M Scotch Magic^®^ tape (3M, Maplewood, MN, USA) was applied onto the electrode laminate side firmly. The peel track was 1.2 cm wide. The Scotch Magic tape was peeled using the top sample holder at a direction of 180° angle to the adhered tape and parallel to one side of the Si electrode. The peeling speed was fixed at 18 cm/min towards the bottom sample holder. The force applied to the adhered tape was recorded during the peeling process. When the tension was fully applied and the electrode laminate was peeled off, the measured force value reaches a plateau, representing the adhesion force of the electrode laminates.

Scanning Electron Microscopy (SEM) images were collected with a JEOL JSM-7500F (JOEL USA, Peabody, MA, USA) field emission scanning electron microscope with an accelerating voltage of 5 kV or 15 kV at room temperature. High-resolution Transmission Electron Microscopy (HRTEM) images were collected on a Philips CM200 (FEI Company, Hillsboro, OR, USA) field emission microscope operated at 200 kV.

All the electrodes and coin cells were prepared and assembled in the Ar-filled glove box. The polymer binders were dissolved in *N*-methyl-2-pyrrolidone (NMP) and then the determined amount of active materials were added. The mixture was mixed by using a homogenizer for 1 h, and the slurry was coated on the copper foil with a doctor blade. The coated electrode was placed in the glove box overnight and further dried in the vacuum oven at 130 °C for 12 h to completely remove the NMP solvents. The electrodes were used to assemble the coin cells. Polypropylene separators Celgard 2400 (Celgard, Charlotte, NC, USA) and the electrolyte (BASF, Independence, OH, USA) consisting of 70%—1.2 M lithium hexafluoro phosphate (LiPF_6_) in ethylene carbonate (EC), diethyl carbonate (DEC) (EC/DEC = 3/7 by weight), and 30%—fluoroethylene carbonate (FEC) by weight were added. As a counter electrode, the Li metal was used. The assembled cells were placed on the channels (Maccor Inc., Tulsa, OK, USA) at 30 °C for lithiation-delithiation cycles. All cells were cycled at a voltage range of 0.01 V–1 V (1C = 1000 mA/g). 

## 3. Results and Discussion

### 3.1. Functional Polymer Binders and Properties

The two polymers are synthesized according to reported procedures with modification (Figure 1a) [19,20]. Esterification of 1-pyrenebutanol and methacryloyl chloride yields the key monomer 1-pyrenebutyl methacrylate, which then undergoes radical polymerization using AIBN to produce the homopolymer PBuPy, as well as the random copolymer PBuPyMAA with methacrylic acid as the co-monomer. The resulting polymers are characterized by NMR and FT-IR to confirm the structure and by GPC to determine the molecular weight. From the proton NMR, it can be clearly viewed that the two singlet for alkenyl protons (δ = 6.09 and 5.54 ppm, Figure S3) in the monomer 1-pyrenebutyl methacrylate disappear after polymerization, indicating the successful synthesis of polymers. For both polymers, a sharp strong peak is found to be around 1725 cm^−1^ in the FT-IR spectra (Figure S1a), which is ascribed to the characteristic C=O stretching. Both polymers exhibit good thermal stability with the onset thermal decomposition temperature at about 350 °C, as shown in the thermal gravimetric analysis (TGA) diagram (Figure S1b).

Benefiting from the molecular design, these polymers serve as effective binders for Si graphene composite particles. The binding mechanism is schematically illustrated in Figure 1d. First, the pyrene units increase the interaction of polymer binders and conductive agent (e.g., graphene) via π-π stacking interaction. At the same time, the carboxyl acid moieties improve the adhesion force of polymer binders with the silicon materials that have plenty of Si-OH groups on the surface. Moreover, the insertion of the flexible butyl segments between polymer backbone and rigid pyrene conjugation units act as a spring in the molecular level and consequently enhance the chain flexibility and increase the polymer free volume, while preserving the conductivity and adhesion functionalities to better serve as binders for silicon containing electrode materials, to maintain the electrode integrity during the drastic volume change of silicon in lithiation and delithiation process.

To demonstrate the function of the butyl group inserted between the pyrene group and the polymer backbone, nano-indentation was conducted on the thin films of pure polymer binders to measure their modulus. PPyMAA [19], which differs from PBuPyMAA by the butyl segment insertion, was also studied. The results are shown in Figure 1b. All the polymers show modulus in the order of 0.1 GPa, much smaller than normal thermoplastic polymer poly(methyl methacrylate) (PMMA, 1.8–3.1 GPa, MIT Material Property Database). It is observed from Figure 1a that the PBuPy and PBuPyMAA exhibit a similar modulus of about 0.35 GPa, smaller than that of PPyMAA (~0.5 GPa), indicating the insertion of butyl segment increases the flexibility of this series of polymers.

On the other hand, pyrene units are expected to furnish the conductive nature of the polymer, while carboxylic acid moieties form ester bond with the hydroxyl groups on Si surface to provide sufficient adhesion [19]. The interaction between the surface Si-OH groups on the surface of Si particles and the carboxylic acid groups on conventional binders such as carboxymethyl cellulose (CMC) or polyacrylic acid (PAA) and its derivatives like PAALi and PAANa plays a very important role in maintaining the adhesion of the binder with the Si material, as well as keeping the mechanical and electronic integrity of the electrode [13,22,23,24,25]. Here, the adhesion force of PBuPy and PBuPyMAA with the Si/graphene material was measured by peel test on the laminates composed of the binder and the Si/graphene material both before and after cycling. The conventional polymer binder PAALi was also studied as a comparison. The peeling track was fixed at 1.2 cm wide and the results were plotted as load vs. distance (N vs. cm) in Figure 1c. Apparently, the conductive polymer binders show similar adhesion properties with PAALi binder at about 5 N loading force for this 1.2 cm wide peeling track and PBuPyMAA, incorporating carboxylic acid group, shows slightly stronger adhesion than that of PBuPy in freshly prepared electrode. After cycling, the cells were disassembled and the cycled electrodes were collected for peel test again. It is clearly viewed that the strong adhesion of the composite electrode is still maintained after long-term cycling with little change compared to that of freshly prepared electrodes.

In total, the PBuPy and PBuPyMAA polymers possess good flexibility and strong adhesive ability, very promising to serve as binders to well accommodate the volume change of silicon materials during charge and discharge.

### 3.2. Morphology of Composite Electrodes before and after Cycling

**Figure 2:**
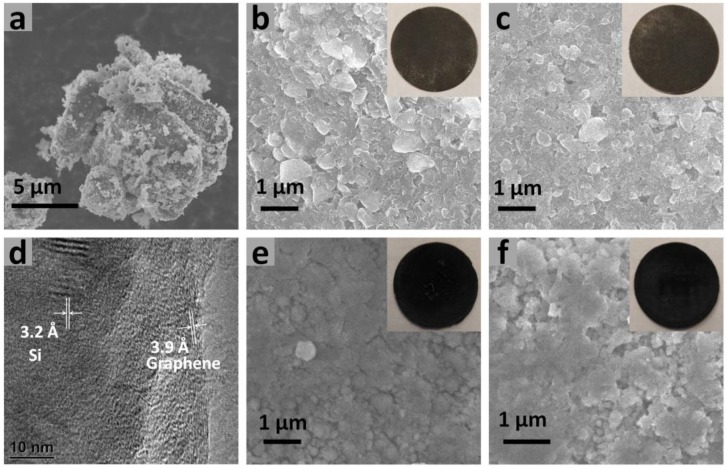
(**a**) Scanning Electron Microscopy (SEM) image of the silicon/graphene material; SEM images of (**b**) the PBuPy electrode and (**c**) the PBuPyMAA electrode before cycling; (**d**) High-resolution Transmission Electron Microscopy (HRTEM) of the silicon/graphene material; SEM images of (**e**) the PBuPy electrode and (**f**) the PBuPyMAA electrode after cycling.

After the successful synthesis of PBuPy and PBuPyMAA, they were used as electrode binders into the electrode matrix with graphene additive and a silicon/graphene composite anode material (48 wt % silicon, obtained from XG Sciences). This silicon/graphene material is synthesized to inherently combine silicon particles with graphene sheets, a novel architecture which helps to maintain the electrical integrity of the electrode against the drastic volume changes during cell cycling. The morphology of these materials, as well as the electrodes is presented in Figure 2.

First, the morphology of the Si/graphene material was studied by scanning electron microscopy (SEM) and high resolution transmission electron microscopy (HRTEM). It is clearly observed from Figure 2a that the composite material particles are about 10 μm in diameter and the surface is furry resulting from the coverage by graphene sheets. This structure is further confirmed by HRTEM (Figure 2d). The outer surface shows graphene patterns with an inter-layer distance of about 3.9 Å and the total thickness of graphene layer is about 5 nm. Meanwhile, crystal silicon phase is found in the inner part of the particle, with a d-spacing about 3.2 Å. The changes in morphology of the composite electrode before and after cycling are also monitored by SEM. Figure 2b,c show the morphology of the freshly prepared PBuPy and PBuPyMAA electrodes before cycling. Both electrodes incorporated with PBuPy and PBuPyMAA polymer binders show tightly packed surface morphology and homogenous distribution of material particles, indicating the use of these binders is appropriate for slurry and lamination process to enable the uniform distribution and close contact between electrode materials, which is beneficial for efficient lithium ion and electron transportation. After cycling, the electrode integrity is still preserved, as exhibited in Figure 2e,f, which show the morphology of the PBuPy electrode and the PBuPyMAA electrode after cycling, respectively. The materials are still homogenously distributed with little cracks found. The above results are consistent with that of Figure 1, suggesting that the PBuPy and PBuPyMAA polymer binders are able to maintain the electrode integrity for long cycle lifetime, due to the flexible polymer chain to accommodate the volume change of silicon material and the strong adhesion to bind the active material particles during cycling.

### 3.3. Electrochemical Perforamnce

The electrodes for cycling test were prepared from the slurry of 10% binder, 80% silicon/graphene material and 10% graphene in NMP, followed by drying in air for 6 h and in vacuum at 130 °C overnight before assembling CR2325-type coin cells. Lithium metal (FMC Corporation, Philadelphia, PA, USA) was used as counter electrode, and a mixture of 70% 1.2 M LiPF_6_ in EC/DEC (3/7, weight ratio) and 30% FEC by weight as electrolyte (EC: ethylene carbonate; DEC: diethyl carbonate; FEC: fluoroethylene carbonate). FEC is well-known to reduce the irreversible capacity of the electrodes and improves the cycling performance with better surface films [26], being able to improve the lifetime of silicon anodes as long as there is sufficient amount of FEC remained during cycling [27,28]. Stable cycling performance was achieved with high capacity and material loading under a high current rate.

**Figure 3:**
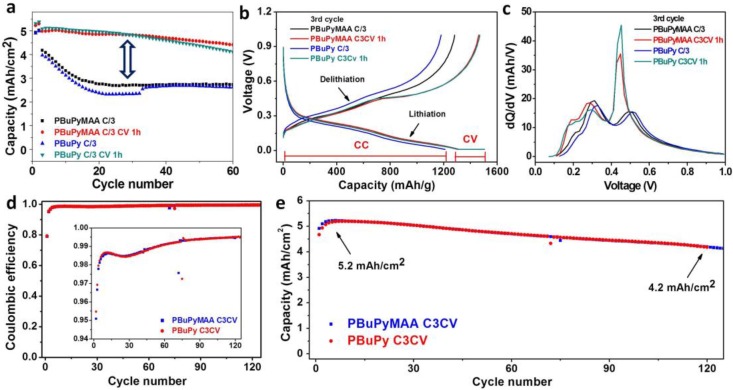
Cycling performance of PBuPy and PBuPyMAA cells. (**a**) areal capacity; (**b**) Voltage-capacity profile at the 3rd cycle; (**c**) dQ/dV vs. voltage diagram of delithiation process at the 3rd cycle of cells under C/3 and C/3 with constant voltage (CV) 1 h procedures; (**d**) coulombic efficiency and (**e**) areal capacity under C/3 with CV till a cut-off current of C/50. (1C = 1 A/g).

The assembled coin cells have considerably high mass loading at around 3.5 mg/cm^2^ and are first cycled under two procedures: (1) C/3 (constant current for both lithiation and delithiation at C/3); (2) C3CV 1 h (lithiation: constant current at C/3 followed by constant voltage at 0.01 V for 1 h, delithiation: constant current at C/3), where 1C = 1 A/g. The two procedures include the same two formation cycles. As shown in Figure 3a, both PBuPy and PBuPyMAA cells show encouraging performance at high mass loading and fast cycling rate with the 1st cycle efficiency close to 80% (Figure S2b). The cells cycled at C/3 show initial capacity loss and fast decay within the first 20 cycles before reaching plateau for both composite electrodes, resulting in an areal capacity of 2.7 mAh/cm^2^ and a gravimetric capacity of 800 mAh/g (Figure S2a), corresponding to 60% capacity retention. However, when running with C3CV 1 h, the cells are showing significantly improved stability and capacity. They exhibit flat cycling curves and retain about 90% capacity after 60 cycles at an areal capacity of 4.4 mAh/cm^2^ and a gravimetric capacity of 1300 mAh/g (Figure S2a). It is presumably owing to the fact that during the CV, the lithiation is at low current rate, enabling more sufficient lithium insertion into the active material and thus higher cycling capacity of the cells. From Figure 3b, it is noted that the CV step contributes a certain amount of the discharge capacity and the charge (delithiation) curves generally show two continuous stages: (1) a gentle slope at 0.1–0.35 V, corresponding to the delithiation of LiC_x_ [4]; and (2) a plateau at 0.35–0.6 V, corresponding to the delithiation of LiSi_x_ [8,9]. It is consistent with the dQ/dV vs. voltage diagram (Figure 3c), which shows three major peaks around 0.2 V, 0.3 V and 0.5 V. The cells with CV step exhibit strong sharp peaks at the silicon region, which account for the major part of the capacity, while that without CV step show weak broad peaks at the Si region and only contribute to a small portion of the capacity, indicating that applying CV process can enhance the utilization of silicon, resulting in higher capacity. While enhanced silicon utilization leads to larger volume change of the active material, it can be accommodated well by the PBuPy and PBuPyMAA polymers, which possess good flexibility and strong adhesive ability, to achieve more stable cycling.

From the above results, it is noticed that applying CV step during lithiation helps to improve the cycling stability and capacity. Therefore, a new cycling procedure with longer CV time was investigated (C3CV, lithiation: constant current at C/3 followed by constant voltage at 0.01 V till a cut-off current of C/50; delithiation: constant current at C/3). Generally, the CV time can last for ~3 h at this procedure, which leads to further improved performance compared to the one with 1 h CV time. The cells show high coulombic efficiency of about 79% at the first cycle and then exceed 99% in the following cycles (Figure 3d). An areal capacity of 5.2 mAh/cm^2^ (Figure 3e) and a volumetric capacity over 1700 mAh/cm^3^ (Figure S2c) have been achieved with 80% retention after 120 cycles, comparatively better than what was reported in the literatures [29,30]. To better evaluate this composite anode, stack energy density was estimated by multiplying volumetric capacity and average delithiation voltage [6,30]. For the PBuPyMAA cell (35% porosity), the stack energy density is calculated to be 918 Wh/L, using a volumetric capacity of 1713 Ah/L and an average delithiation voltage of 0.4768 V, while that for the PBuPy cell (34% porosity) is calculated to be 915 Wh/L, using a volumetric capacity of 1695 Ah/L and an average delithiation voltage of 0.4821 V, corresponding to a significant increase of about 26% in energy density compared to a LiCoO_2_/graphite cell (726 Wh/L) [6].

Furthermore, the rate performance of the cells (Figure S2d) indicates that the two polymer binders are able to deliver high capacity and stable cycling under moderately high current rate for the silicon/graphene material. Increasing the current rate from C/10 to C/5 only leads to about 15% capacity loss and 50% capacity is retained even at 1C current rate. When the current rate is increased to the higher region, the capacity loss is larger. However, the capacity can fully recover when the current returns to a low rate at C/10, implying that the electrode material is stable and the electrode integrity is well maintained after fast charge/discharge.

## 4. Conclusions

In summary, two novel polymer binders—PBuPy and PBuPyMAA—with tunable functionalities have been developed to effectively facilitate the electrochemical performance of Si containing anodes in lithium ion batteries. Nano-indentation and peel test exhibit the improved flexibility of the two novel conductive polymer binders and their high adhesion force with Si containing active material, both of which are beneficial to maintain the electrode integrity. High mass loading of active material has been achieved for both binders with a silicon/graphene composite material, showing remarkably high capacity and cycling stability. An area capacity of over 5 mAh/cm^2^ and volumetric capacity of over 1700 Ah/L have been achieved at high current rate of 333 mA/g with potentiostatic step during lithiation. The results indicate that these polymer binders could be promising for the practical application of silicon materials in lithium ion batteries.

## Supplementary Materials

The following are available online at www.mdpi.com/2073-4360/9/12/657/s1, Figure S1: TGA curve of (a) PBuPy and (b) PBuPyMAA; Figure S2: (a) Gravimetric capacity; (b) Coulombic efficiency of the cells with PBuPy and PBuPyMAA binders under C/3 and C3CV 1 h cycling procedures; (c) volumetric capacity and gravimetric capacity of the cells with PBuPy and PBuPyMAA binders under C3CV cycling procedure; (d) rate test of the cells with PBuPy and PBuPyMAA binders; Figure S3: ^1^H NMR spectrum of 1-Pyrenebutyl methacrylate; Figure S4: MALDI-TOF spectrum of 1-Pyrenebutyl methacrylate; Figure S5: ^1^H NMR spectrum of PBuPy; Figure S6: ^1^H NMR spectrum of PBuPyMAA.
