# Supplementary material for: Molecular Spring Enabled High-Performance Anode for Lithium Ion Batteries
Source: Polymers (Basel). 2017 Nov 29;9(12):657. doi: 10.3390/polym9120657 (PMC6418860; doi:10.3390/polym9120657)
Supplement: Supplementary file 1 [file polymers-09-00657-s001.pdf]

Molecular Spring Enabled High-performance Silicon/graphene Composite Anode in Lithium Ion Batteries

Tianyue Zheng, Zhe Jia, Na Lin, Thorsten Langer,Simon Lux, Isaac Lund,Ann-Christin Gentschev, Juan Qiao, and Gao Liu*

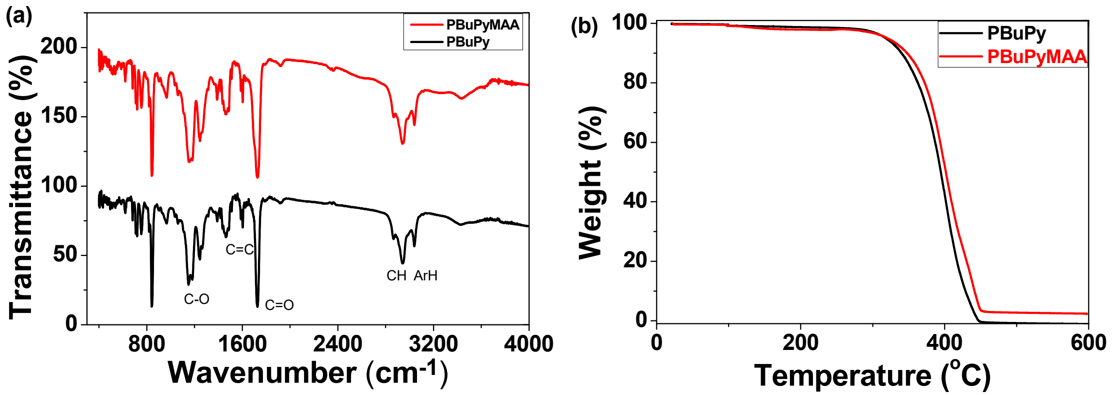

Figure S1. (a) FT-IR spectra, (b) TGA curve of PBuPy and PBuPyMAA

Thermogravimetric analysis (TGA) allows the measurement of polymer thermal stability, measured in nitrogen flow with temperatures ramped up to 600 °C. Both polymers exhibit a good thermal stability with decomposition temperature at about 350 °C.

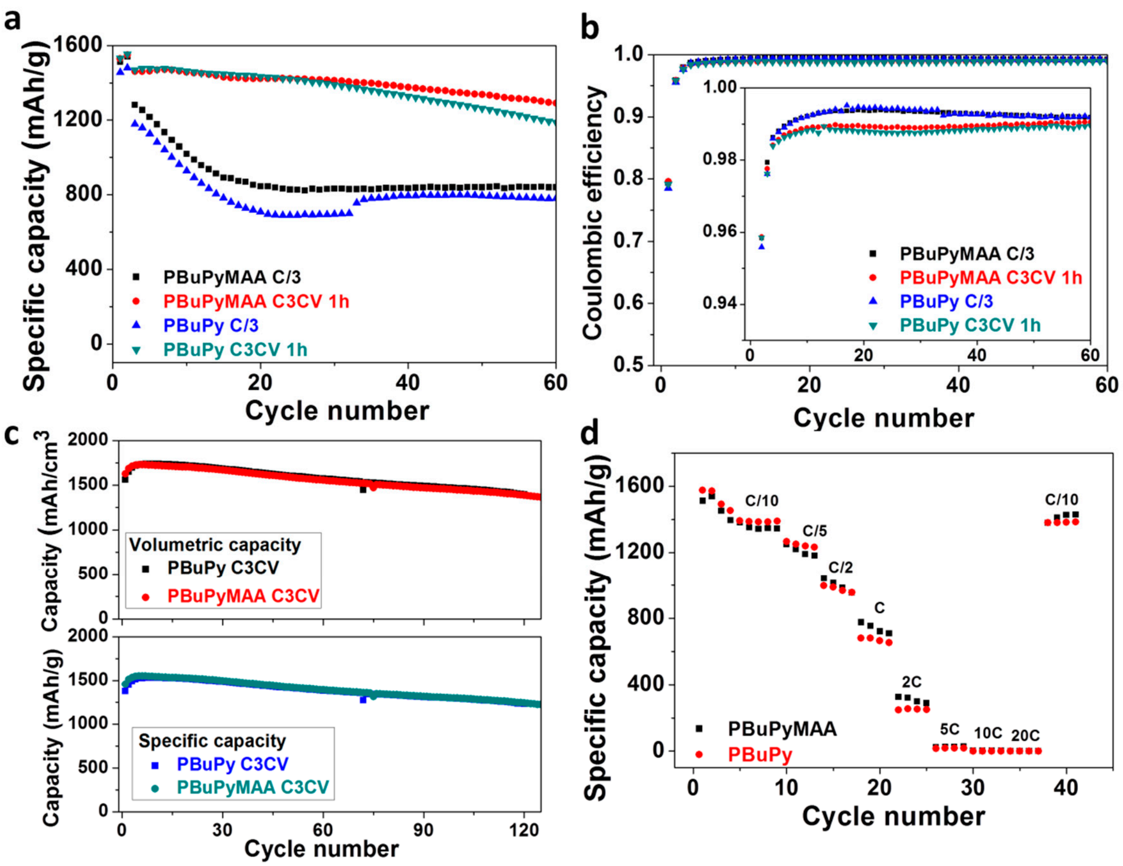

Figure S2. (a) Gravimetric capacity (b) Coulombic efficiency of the cells with PBuPy and PBuPyMAA binders under C/3 and C3CV 1h cycling procedures, (c) volumetric capacity and gravimetric capacity of the cells with PBuPy and PBuPyMAA binders under C3CV cycling procedure, (d) rate test of the cells with PBuPy and PBuPyMAA binders.

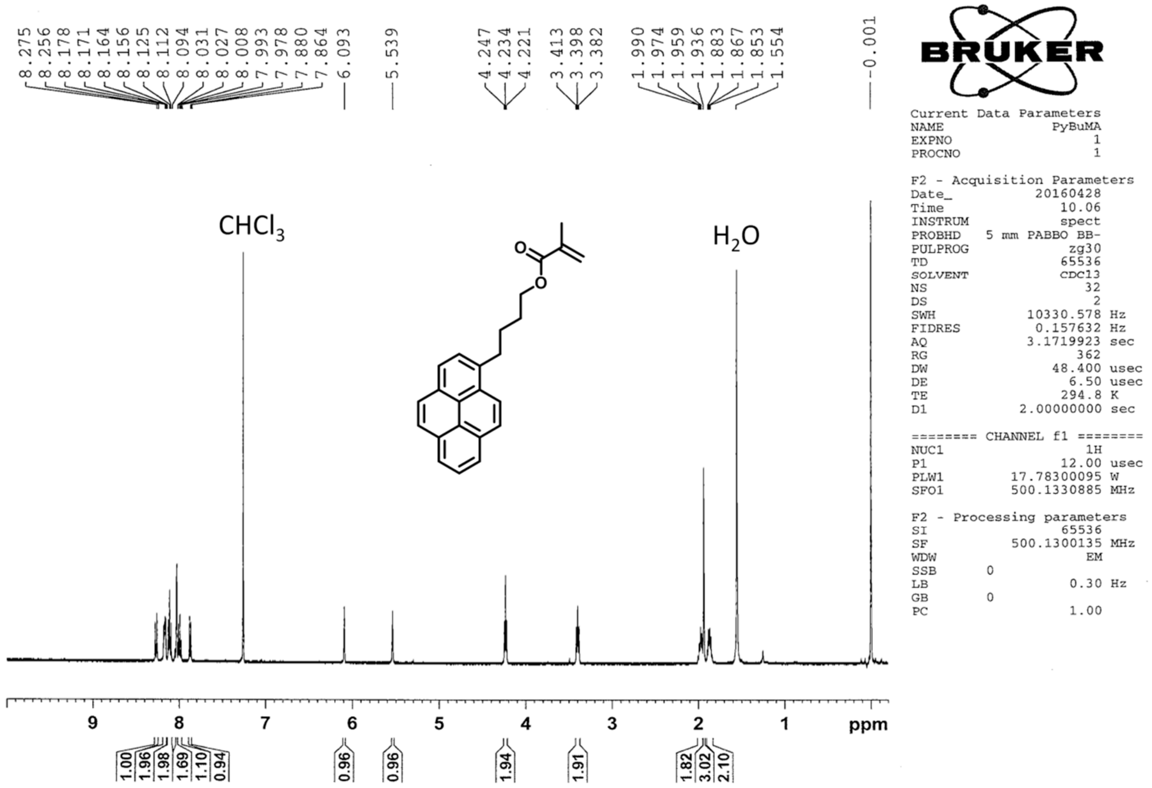

Figure S3. ^{1}H-NMR spectrum of 1-Pyrenebutyl methacrylate.

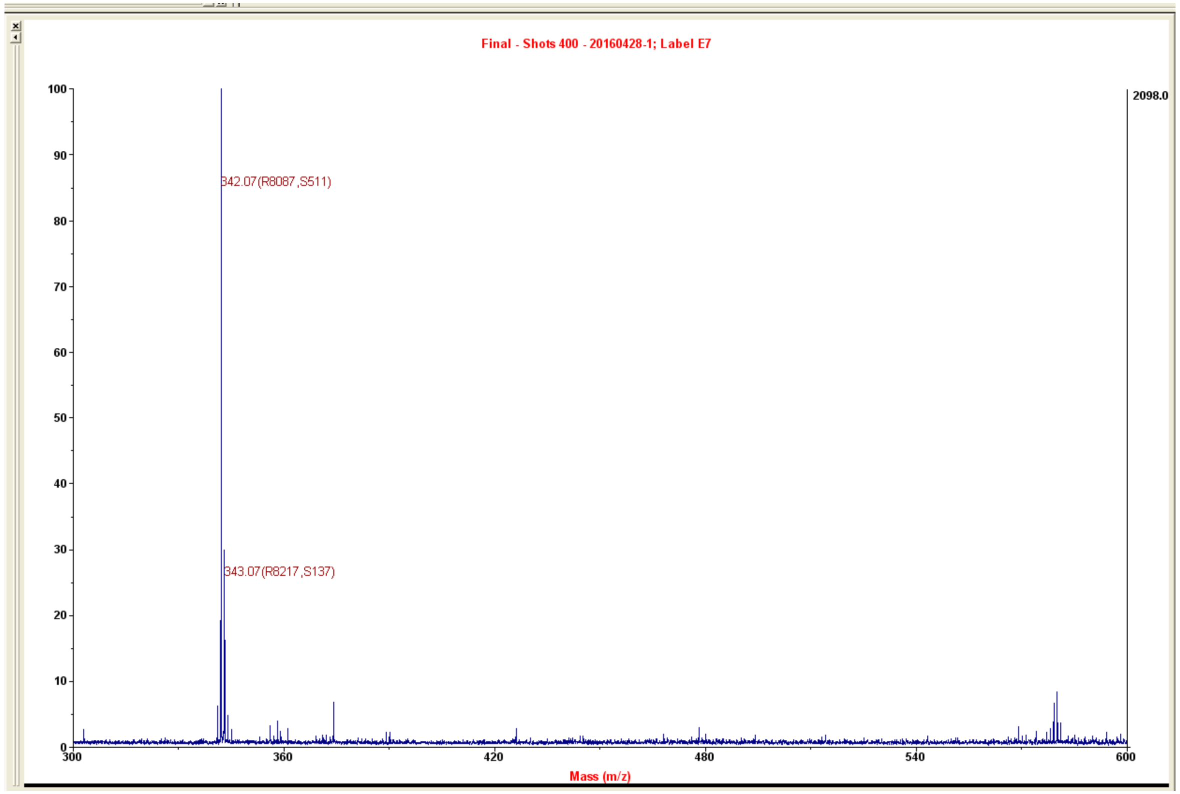

Figure S4. MALDI-TOF spectrum of 1-Pyrenebutyl methacrylate.

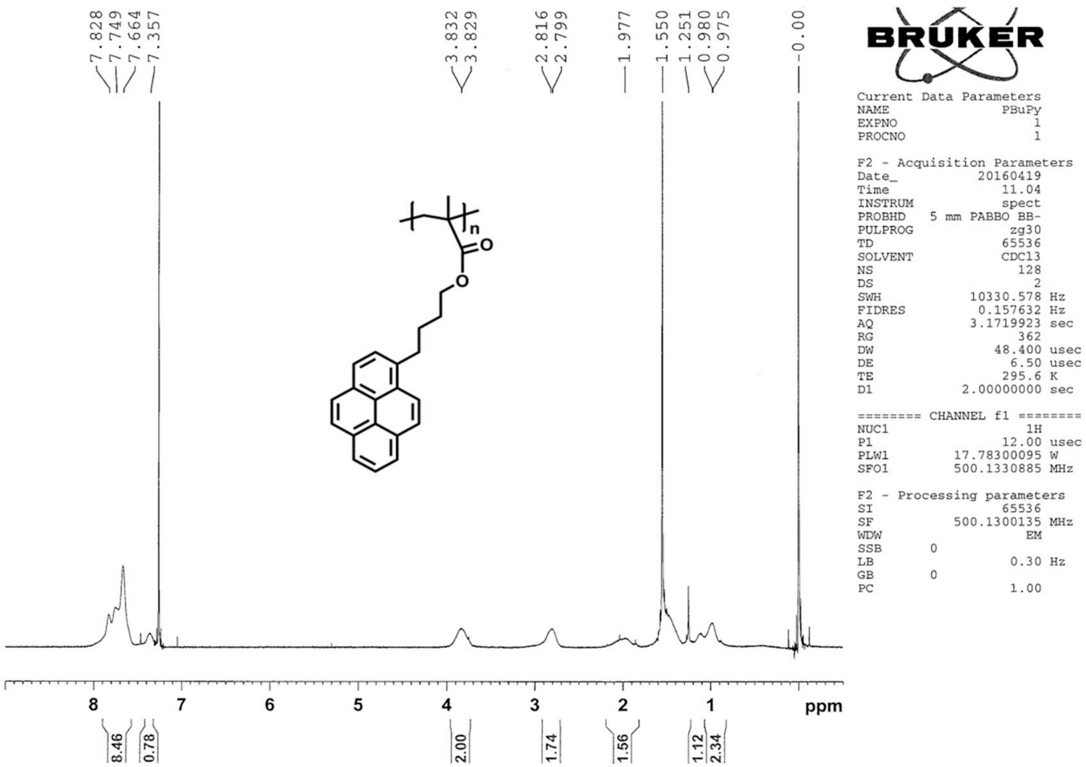

Figure S5. ^{1}H-NMR spectrum of PBuPy.

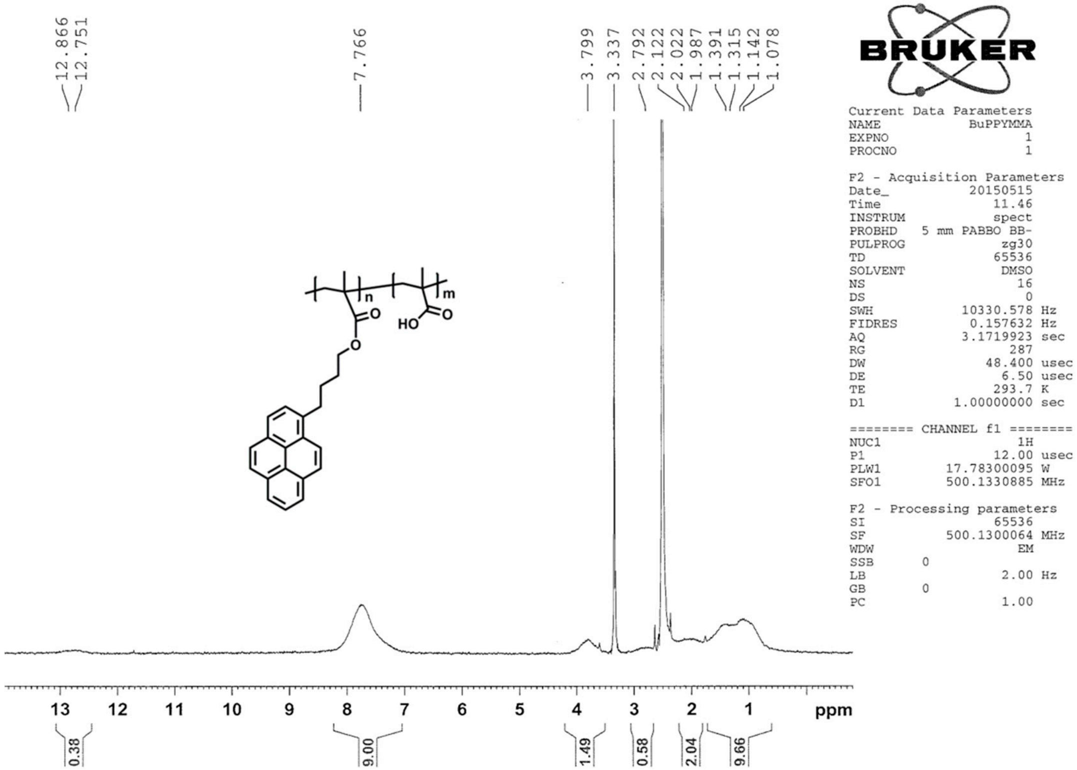

Figure S6. ^{1}H-NMR spectrum of PBuPyMAA.
